# Supplementary material for: Genomic investigation of a dengue virus outbreak in Thiès, Senegal, in 2018
Source: Sci Rep. 2021 May 14;11:10321. doi: 10.1038/s41598-021-89070-1 (PMC8121849; doi:10.1038/s41598-021-89070-1)
Supplement: Supplementary file 4 — Supplemental File 3. [file 41598_2021_89070_MOESM4_ESM.pdf]

Supplementary Materials

Genomic investigation of a dengue virus outbreak in Thiès, Senegal, in 2018

Amy Gaye, Tolla Ndiaye, Mouhamad Sy, Awa B. Deme, Alphonse B. Thiaw, Aita Sene, Cheikh Ndiaye, Younouss Diedhiou, Amadou M. Mbaye, Ibrahima Ndiaye, Christopher Tomkins-Tinch, Jules F. Gomis, Aida S. Badiane, Bronwyn MacInnis, Daniel J. Park, Mouhamadou Ndiaye, Ngayo Sy, Pardis C. Sabeti, Katherine J. Siddle, Daouda Ndiaye

Figure S1. Heatmap of normalized counts for viral taxa from the metagenomic sequencing data using KrakenUniq. Shown are the viral species known to infect humans for which at least 1 of the 18 sequenced samples identified as positive for dengue virus had >100 reads per million sequenced reads assigned to that species. Bacteriophages have been removed. Kyasanur forest disease virus, Powassan virus and Sepik virus are all flaviviruses, suggesting these may be misclassifications of reads within this large viral genus. Human associated gemykibivirus 2 is a virus of unknown pathogenic effect in humans.

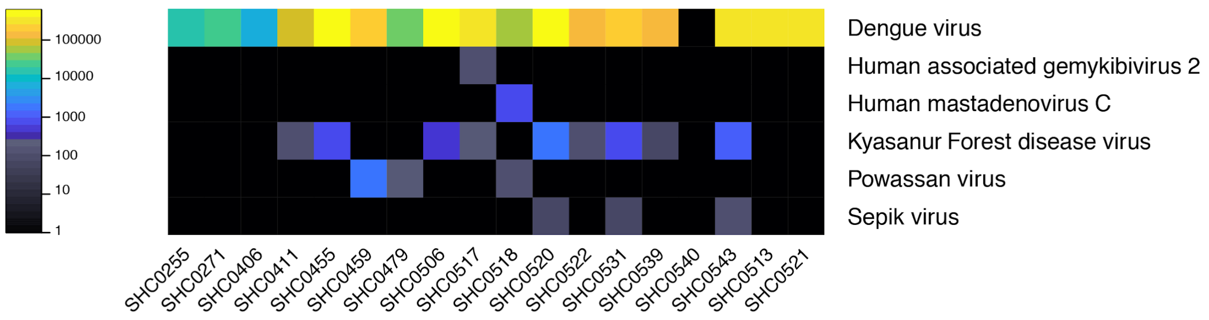

Figure S2. Alignment of select dengue virus genomes to pan-flavivirus primer. DENV1 sequences from Senegal (SHC0513 and SHC0521) showed presence of 4 mismatches (C8941T, C8950T, G8953A and G8962A) in the primer binding region of pan-flavivirus forward primer (in blue). This likely explains why these sequences were not detected by this assay, unlike DENV2 and DENV3.

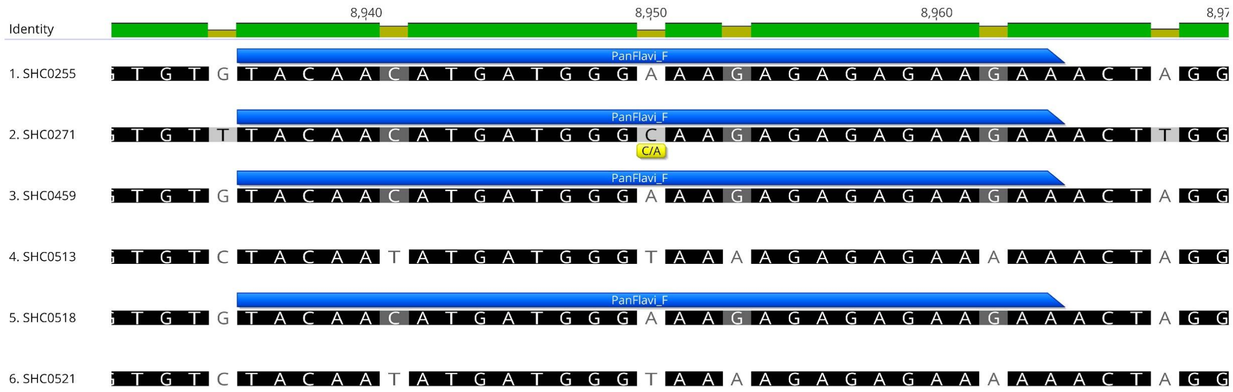

Figure S3. Maximum Likelihood tree of a 955nt fragment of the envelope gene of DENV3 showing the relationship between available African sequences. Tips are coloured by country and GenBank IDs are given for available African sequences. All non-African sequences are gray. Bootstrap values are indicated on major nodes associated with African sequences.

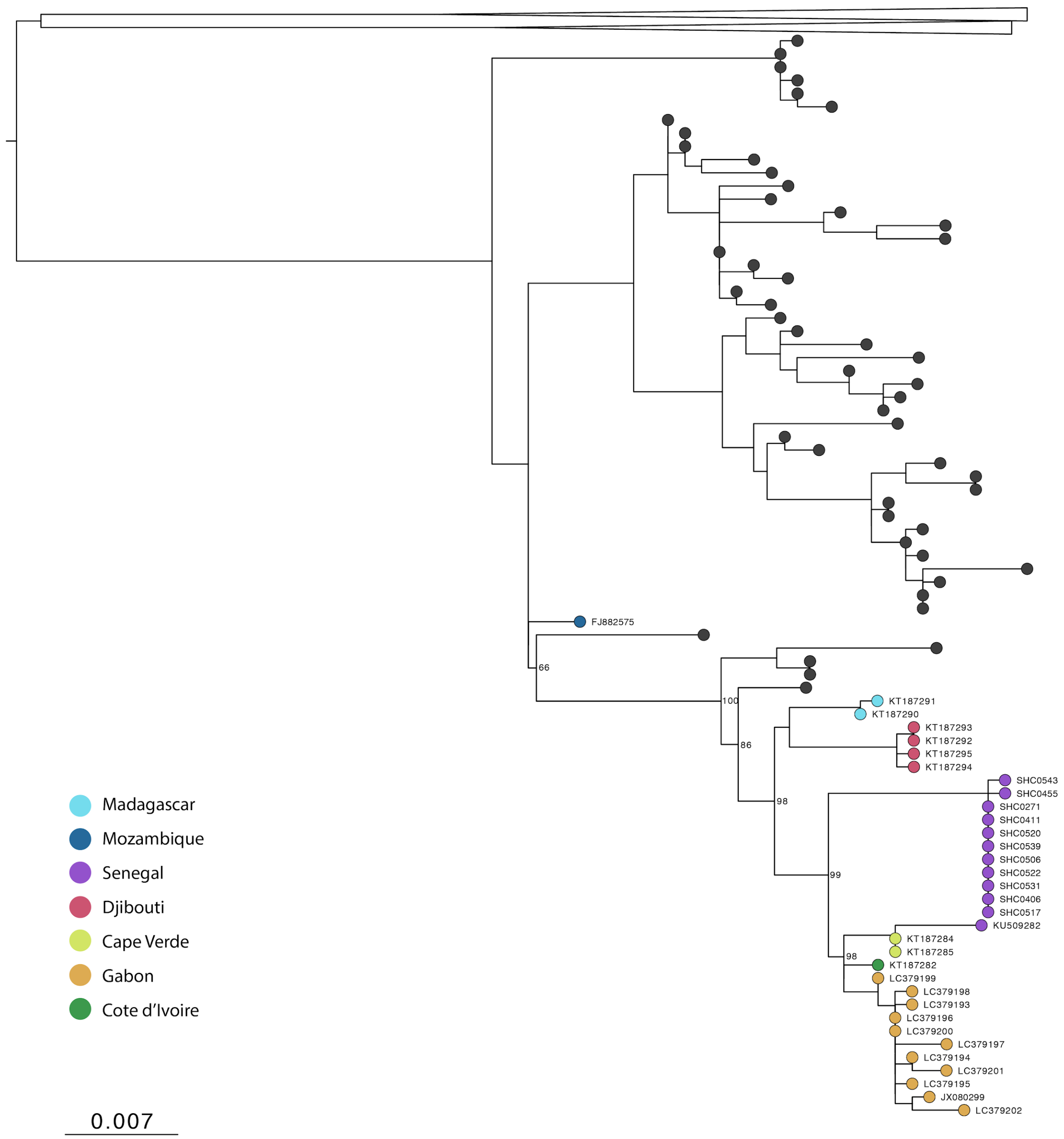

Figure S4. Maximum Likelihood tree of a 245nt fragment of the polymerase gene for 60 sequences from DENV1 genotype III. A single randomly selected sequence from each other genotype (I, II, IV and V) are included as outgroups. The new sequences from Senegal (SHC0521 and SHC0513) cluster with other sequences from elsewhere in West Africa and are distinct from those from Senegal collected in 2015 (MK940790 and MK940791).

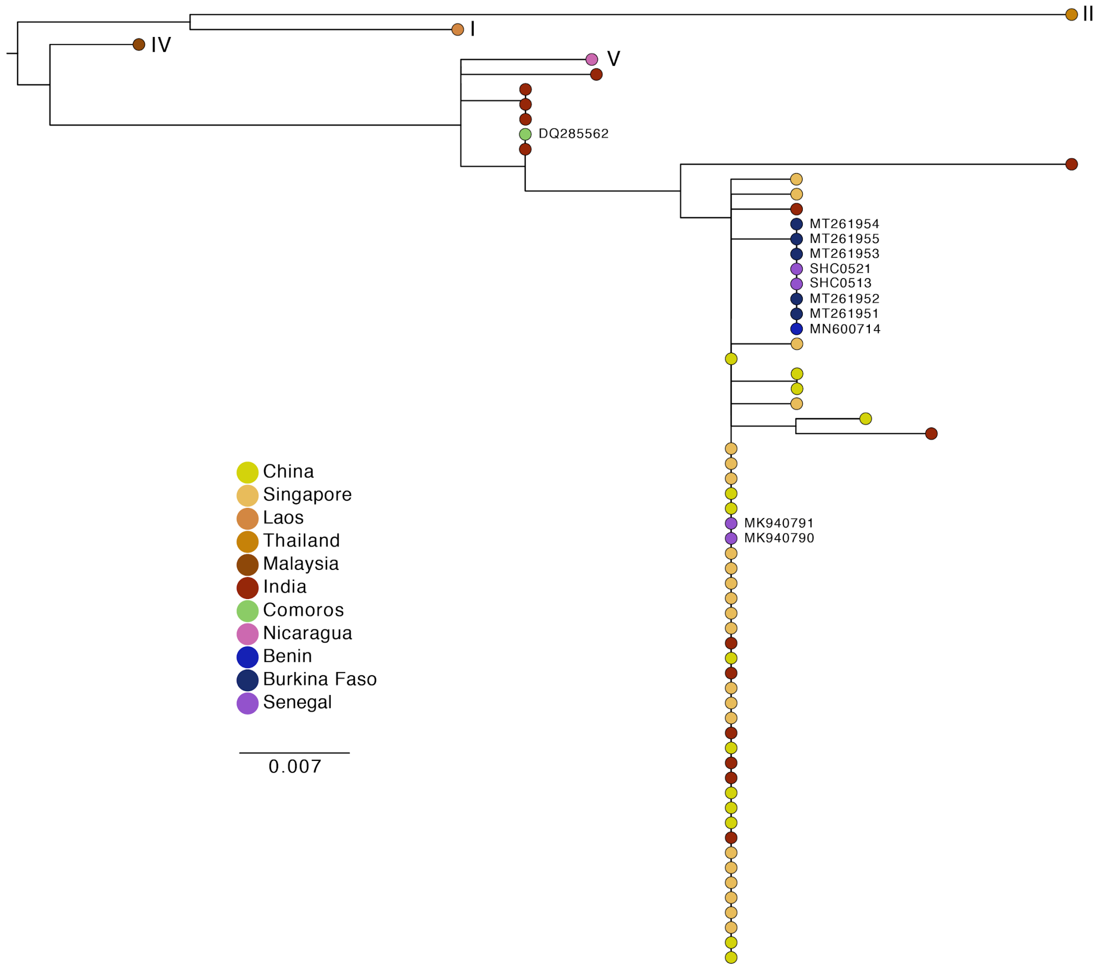

Table S1. Sequencing metrics for samples in the study in which dengue virus was detected by RT-qPCR or sequencing.

Sample ID	CT mean	Total read pairs	Total mapped read pairs	Assembled genome length	Percentage of the reference genome covered	Mean coverage across the reference genome	Virus serotype
SHC0255	33.04	3127961	2832555	10659	99.98	456.9	DENV2
SHC0271	34.3	242086	233966	10513	100	18.3	DENV3
SHC0406	35.12	1240460	821230	10525	100	28.6	DENV3
SHC0411	32.85	1508912	1436468	10655	99.98	621.4	DENV3
SHC0455	28.91	2112154	2022073	10680	100	5366.1	DENV3
SHC0459	30.17	81572	51926	10640	100	147.1	DENV2
SHC0479	30.55	1114283	1039515	10641	100	168.3	DENV2
SHC0503	34.1	#N/A	#N/A	#N/A	#N/A	#N/A	#N/A
SHC0506	25.86	2235109	2022819	10680	100	4127.5	DENV3
SHC0513	#N/A	1568767	2566696	10693	100	3896.1	DENV1
SHC0517	24.63	1928183	1816047	10650	99.98	1807.7	DENV3
SHC0518	27.45	216660	213657	10551	100	34.8	DENV2
SHC0520	24.66	503591	412026	10641	100	101	DENV3
SHC0521	#N/A	1181020	1999070	10683	100	3156	DENV1
SHC0522	29.42	1397584	1313920	10663	100	512.4	DENV3
SHC0531	28.2	863473	602554	10639	100	370.3	DENV3
SHC0539	23.6	3047434	2754428	10662	100	1601.8	DENV3
SHC0540	36.01	2439616	2211767	#N/A	#N/A	#N/A	#N/A
SHC0543	26.9	1002324	713170	10650	100	640.7	DENV3

Additional data files

XML files used for BEAST analysis of complete genomes from each serotype. These files are provided as 3 separate files:

Supplemental File 1: XML file for BEAST computation for dengue 1

Supplemental File 2: XML file for BEAST computation for dengue 2

Supplemental File 3: XML file for BEAST computation for dengue 3
